# (5*RS*,10*SR*,15*RS*)-Trimethyl­truxene^1^

**DOI:** 10.1107/S1600536811048616

**Published:** 2011-11-30

**Authors:** Kandace R. Thomas, Raj K. Dhar, Frank R. Fronczek, Steven F. Watkins

**Affiliations:** a Department of Chemistry, Louisiana State University, Baton Rouge, LA 70803-1804, USA

## Abstract

The title mol­ecule, C_30_H_24_, was prepared as a possible precursor to buckminsterfullerene cages. The two enanti­omers adopt the *anti* configuration, with one *S*/*R* and two *R*/*S* methyl groups, one *anti* to the other two. The truxene framework is slightly non-planar: with respect to the central six-ring mean plane, the three methyl C atoms are 1.377 (3), −1.475 (3) and 1.515 (3) Å distant, whereas the respective proximate peripheral six-ring mean planes make dihedral angles of 6.27 (6), 3.45 (7) and −7.37 (7)°.

## Related literature

For related structures, see: De Frutos *et al.* (1999 ▶, 2002 ▶). For the synthesis of truxenes, see: Amick & Scott (2007 ▶); Dehmlow & Kelle (1997 ▶); Kipping (1894*a* ▶,*b* ▶); Hausmann (1889 ▶); Wislicenus (1887 ▶). For buckminsterfullerene, see: Kroto *et al.* (1985 ▶). Buckybowls are inter­mediates in the synthesis of buckminsterfullerene. Truxene compounds, which serve as backbone of bucky bowl derivatives, have been fabricated for use as star-shaped organic semiconductors in solution, see: Sun *et al.* (2005 ▶).
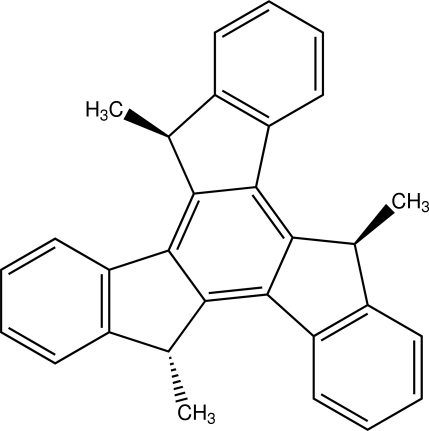


## Experimental

### 

#### Crystal data

C_30_H_24_
*M* *_r_* = 384.49
Monoclinic, 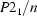

*a* = 8.6755 (2) Å
*b* = 18.2860 (4) Å
*c* = 12.8206 (3) Å
β = 96.007 (1)°
*V* = 2022.69 (8) Å^3^
*Z* = 4
Mo *K*α radiation
μ = 0.07 mm^−1^
*T* = 90 K
0.15 × 0.15 × 0.13 mm

#### Data collection

Nonius KappaCCD diffractometer
Absorption correction: multi-scan (*SCALEPACK*; Otwinowski & Minor, 1997 ▶) *T*_min_ = 0.955, *T*_max_ = 0.976
13386 measured reflections
7338 independent reflections
5008 reflections with *I* > 2σ(*I*)
*R*_int_ = 0.034

#### Refinement

*R*[*F* ^2^ > 2σ(*F* ^2^)] = 0.053
*wR*(*F* ^2^) = 0.157
*S* = 1.04
7338 reflections
274 parameters
H-atom parameters constrained
Δρ_max_ = 0.48 e Å^−3^
Δρ_min_ = −0.29 e Å^−3^

### 

Data collection: *COLLECT* (Nonius, 2000 ▶); cell refinement: *SCALEPACK* (Otwinowski & Minor, 1997 ▶); data reduction: *DENZO* (Otwinowski & Minor, 1997 ▶) and *SCALEPACK*; program(s) used to solve structure: *SHELXS86* (Sheldrick, 2008 ▶); program(s) used to refine structure: *SHELXL97* (Sheldrick, 2008 ▶); molecular graphics: *ORTEP-3 for Windows* (Farrugia, 1997 ▶); software used to prepare material for publication: *WinGX* (Farrugia, 1999 ▶).

## Supplementary Material

Crystal structure: contains datablock(s) global, I. DOI: 10.1107/S1600536811048616/qm2041sup1.cif

Structure factors: contains datablock(s) I. DOI: 10.1107/S1600536811048616/qm2041Isup2.hkl

Additional supplementary materials:  crystallographic information; 3D view; checkCIF report

## Figures and Tables

**Fig. 1.**
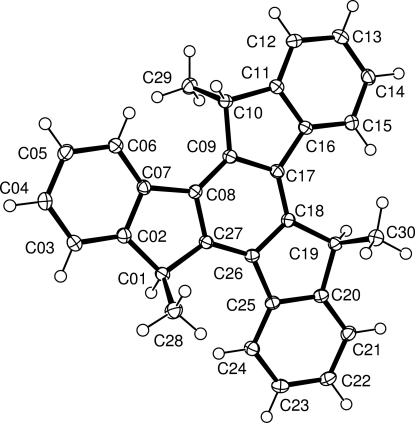
View of (I) (50% probability displacement ellipsoids)

## supplementary crystallographic information

### Comment

Buckminsterfullerene is a spherical fullerene molecule with the formula C_60_. It was first prepared in 1985 by Kroto *et al.* Buckybowls have been recognized as valuable intermediates in the synthesis of buckminsterfullerene and also show many valuable and interesting properties, including surface selective chemistry. As a part of ongoing investigations for synthesis of bucky bowls, the title molecule, trimethyltruxene, C_30_H_24_, was prepared as a possible precursor to buckminsterfullerene cages. Furthermore, these truxene compounds, which serve as backbone of bucky bowl derivatives also have shown properties of organic field-effect transistors (OFETs) based on oligothiophene-functionalized truxene derivatives, which have been fabricated for use as novel star-shaped organic semiconductors in solution (Sun *et al.*, 2005).

Two isomers of the title compound, *syn* and *anti* are possible. De Frutos *et al.* (2002) prepared mixtures of the two isomers, which could be converted to the pure, more stable *syn* compound by reaction with base, potassium t-butoxide. We report here the structure of the less stable *anti* isomer, which is not a viable precursor for buckybowls.

The parent heptacyclic aromatic system, truxene (10,15-dihydro-5*H*-diindeno[1,2 - *a*:1',2'-*c*]fluorene), is planar with 3/m (C_3 h_) symmetry. The title molecule, I, is slightly non-planar with no discernable pattern: with respect to the central 6-ring mean plane, the three methyl groups are +1.377 (3), -1.475 (3) and +1.515 (3) Å distant, whereas the proximate peripheral 6-ring mean planes make dihedral angles of +6.27 (6)°, +3.45 (7)° and -7.37 (7)°.

### Experimental

Synthesis of truxene was carried by the one-pot, acid catalyzed, head-to-tail cyclotrimerization synthesis method detailed in (Amick & Scott, 2007) The trimethylation of truxene was carried out by treating truxene with *n*-butyl lithium, followed by treatment with methyl iodide. A suitable single-crystal was obtained by recrystallization from diethyl ether, dichloromethane and methanol.

### Refinement

All H atoms were placed in calculated positions, guided by difference maps, with C—H bond distances 0.95 (aromatic C), 1.00 (alkyl C) Å, and *U*_iso_=1.2*U*_eq_, thereafter refined as riding. A torsional parameter was refined for each methyl group, with C—H bond distances 0.98 Å and *U*_iso_ = 1.5*U*_eq_. The largest peak in the final difference map was at the center of the C18–C26 bond, and the top 33 peaks lay near bond centers.

### Crystal data

**Table:**

C_30_H_24_	*F*(000) = 816
*M**_r_* = 384.49	*D*_x_ = 1.263 Mg m^−^^3^
Monoclinic, *P*2_1_/*n*	Mo *K*α radiation, λ = 0.71073 Å
Hall symbol: -P 2yn	Cell parameters from 6571 reflections
*a* = 8.6755 (2) Å	θ = 2.5–33.5°
*b* = 18.2860 (4) Å	µ = 0.07 mm^−^^1^
*c* = 12.8206 (3) Å	*T* = 90 K
β = 96.007 (1)°	Prism, yellow
*V* = 2022.69 (8) Å^3^	0.15 × 0.15 × 0.13 mm
*Z* = 4	

### Data collection

**Table:**

Nonius KappaCCD diffractometer	7338 independent reflections
Radiation source: sealed tube	5008 reflections with *I* > 2σ(*I*)
graphite	*R*_int_ = 0.034
Detector resolution: 9 pixels mm^-1^	θ_max_ = 33.5°, θ_min_ = 2.6°
CCD rotation images, thick slices scans	*h* = −13→13
Absorption correction: multi-scan (*SCALEPACK*; Otwinowski & Minor, 1997)	*k* = −28→23
*T*_min_ = 0.955, *T*_max_ = 0.976	*l* = −19→19
13386 measured reflections	

### Refinement

**Table:**

Refinement on *F*^2^	Primary atom site location: structure-invariant direct methods
Least-squares matrix: full	Secondary atom site location: difference Fourier map
*R*[*F*^2^ > 2σ(*F*^2^)] = 0.053	Hydrogen site location: inferred from neighbouring sites
*wR*(*F*^2^) = 0.157	H-atom parameters constrained
*S* = 1.04	*w* = 1/[σ^2^(*F*_o_^2^) + (0.0893*P*)^2^ + 0.0548*P*] where *P* = (*F*_o_^2^ + 2*F*_c_^2^)/3
7338 reflections	(Δ/σ)_max_ = 0.001
274 parameters	Δρ_max_ = 0.48 e Å^−^^3^
0 restraints	Δρ_min_ = −0.29 e Å^−^^3^
0 constraints	

### Special details

**Table:**

Geometry. All e.s.d.'s (except the e.s.d. in the dihedral angle between two l.s. planes) are estimated using the full covariance matrix. The cell e.s.d.'s are taken into account individually in the estimation of e.s.d.'s in distances, angles and torsion angles; correlations between e.s.d.'s in cell parameters are only used when they are defined by crystal symmetry. An approximate (isotropic) treatment of cell e.s.d.'s is used for estimating e.s.d.'s involving l.s. planes.

### Fractional atomic coordinates and isotropic or equivalent isotropic displacement parameters (Å^2^)

**Table:**

	*x*	*y*	*z*	*U*_iso_*/*U*_eq_	
C01	−0.07933 (13)	0.09157 (6)	0.49364 (9)	0.0170 (2)	
H01	−0.0475	0.0572	0.5526	0.020*	
C02	−0.19146 (13)	0.05432 (6)	0.41175 (10)	0.0174 (2)	
C03	−0.33977 (14)	0.02847 (7)	0.42222 (10)	0.0203 (2)	
H03	−0.3798	0.0296	0.4884	0.024*	
C04	−0.42872 (14)	0.00091 (7)	0.33434 (10)	0.0206 (2)	
H04	−0.5294	−0.0178	0.3409	0.025*	
C05	−0.37157 (14)	0.00050 (7)	0.23706 (10)	0.0192 (2)	
H05	−0.4346	−0.0173	0.1774	0.023*	
C06	−0.22251 (13)	0.02602 (6)	0.22615 (10)	0.0177 (2)	
H06	−0.1835	0.0254	0.1596	0.021*	
C07	−0.13174 (13)	0.05235 (6)	0.31430 (9)	0.0154 (2)	
C08	0.02504 (13)	0.08458 (6)	0.32688 (9)	0.0153 (2)	
C09	0.13369 (13)	0.09525 (6)	0.25572 (9)	0.0152 (2)	
C10	0.13230 (13)	0.06937 (6)	0.14305 (9)	0.0172 (2)	
H10	0.0400	0.0894	0.0990	0.021*	
C11	0.28051 (14)	0.10304 (6)	0.11113 (10)	0.0174 (2)	
C12	0.34197 (15)	0.09751 (7)	0.01588 (10)	0.0213 (3)	
H12	0.2860	0.0734	−0.0418	0.026*	
C13	0.48723 (15)	0.12793 (7)	0.00611 (10)	0.0223 (3)	
H13	0.5300	0.1249	−0.0590	0.027*	
C14	0.57001 (14)	0.16264 (6)	0.09067 (10)	0.0197 (2)	
H14	0.6696	0.1823	0.0832	0.024*	
C15	0.50823 (14)	0.16888 (6)	0.18647 (10)	0.0182 (2)	
H15	0.5647	0.1928	0.2441	0.022*	
C16	0.36210 (13)	0.13934 (6)	0.19621 (9)	0.0154 (2)	
C17	0.27069 (13)	0.13486 (6)	0.28678 (9)	0.0149 (2)	
C18	0.30010 (12)	0.16021 (6)	0.38920 (9)	0.0148 (2)	
C19	0.43313 (13)	0.20650 (6)	0.43845 (9)	0.0165 (2)	
H19	0.5346	0.1829	0.4286	0.020*	
C20	0.40577 (13)	0.20572 (6)	0.55330 (9)	0.0162 (2)	
C21	0.49513 (13)	0.23644 (7)	0.63774 (10)	0.0191 (2)	
H21	0.5885	0.2614	0.6278	0.023*	
C22	0.44625 (14)	0.23020 (7)	0.73774 (10)	0.0204 (2)	
H22	0.5061	0.2515	0.7962	0.025*	
C23	0.30980 (14)	0.19282 (7)	0.75213 (10)	0.0197 (2)	
H23	0.2784	0.1884	0.8206	0.024*	
C24	0.21912 (13)	0.16198 (6)	0.66747 (9)	0.0178 (2)	
H24	0.1268	0.1363	0.6779	0.021*	
C25	0.26576 (13)	0.16933 (6)	0.56710 (9)	0.0153 (2)	
C26	0.19633 (13)	0.14324 (6)	0.46379 (9)	0.0148 (2)	
C27	0.05726 (13)	0.10782 (6)	0.43211 (9)	0.0149 (2)	
C28	−0.15190 (14)	0.16121 (7)	0.53548 (11)	0.0226 (3)	
H28A	−0.0753	0.1860	0.5849	0.034*	
H28B	−0.2422	0.1479	0.5714	0.034*	
H28C	−0.1843	0.1940	0.4768	0.034*	
C29	0.13709 (15)	−0.01460 (7)	0.13416 (11)	0.0230 (3)	
H29A	0.1441	−0.0285	0.0610	0.034*	
H29B	0.0425	−0.0354	0.1578	0.034*	
H29C	0.2277	−0.0334	0.1781	0.034*	
C30	0.42829 (15)	0.28518 (7)	0.39498 (10)	0.0223 (3)	
H30A	0.4338	0.2838	0.3190	0.033*	
H30B	0.5166	0.3129	0.4286	0.033*	
H30C	0.3315	0.3088	0.4097	0.033*	

### Atomic displacement parameters (Å^2^)

**Table:**

	*U*^11^	*U*^22^	*U*^33^	*U*^12^	*U*^13^	*U*^23^
C01	0.0155 (5)	0.0216 (5)	0.0142 (5)	−0.0022 (4)	0.0025 (4)	0.0001 (4)
C02	0.0162 (5)	0.0190 (5)	0.0171 (6)	−0.0013 (4)	0.0021 (4)	0.0002 (4)
C03	0.0189 (5)	0.0244 (6)	0.0184 (6)	−0.0038 (5)	0.0051 (5)	−0.0007 (5)
C04	0.0163 (5)	0.0235 (6)	0.0224 (6)	−0.0043 (5)	0.0036 (5)	−0.0006 (5)
C05	0.0167 (5)	0.0208 (6)	0.0196 (6)	−0.0017 (4)	−0.0004 (4)	−0.0024 (5)
C06	0.0165 (5)	0.0205 (5)	0.0162 (6)	−0.0013 (4)	0.0016 (4)	−0.0009 (4)
C07	0.0137 (5)	0.0161 (5)	0.0164 (5)	−0.0005 (4)	0.0022 (4)	−0.0001 (4)
C08	0.0144 (5)	0.0170 (5)	0.0143 (5)	−0.0010 (4)	0.0007 (4)	−0.0001 (4)
C09	0.0147 (5)	0.0172 (5)	0.0139 (5)	0.0002 (4)	0.0019 (4)	0.0004 (4)
C10	0.0160 (5)	0.0215 (5)	0.0143 (5)	−0.0022 (4)	0.0023 (4)	−0.0018 (4)
C11	0.0177 (5)	0.0193 (5)	0.0155 (5)	−0.0012 (4)	0.0035 (4)	−0.0004 (4)
C12	0.0229 (6)	0.0254 (6)	0.0156 (6)	−0.0037 (5)	0.0026 (5)	−0.0035 (5)
C13	0.0246 (6)	0.0269 (6)	0.0168 (6)	−0.0022 (5)	0.0080 (5)	−0.0001 (5)
C14	0.0190 (5)	0.0197 (5)	0.0214 (6)	−0.0024 (4)	0.0065 (5)	0.0001 (5)
C15	0.0174 (5)	0.0193 (5)	0.0183 (6)	−0.0018 (4)	0.0032 (4)	−0.0004 (4)
C16	0.0158 (5)	0.0166 (5)	0.0142 (5)	0.0005 (4)	0.0033 (4)	0.0003 (4)
C17	0.0141 (5)	0.0165 (5)	0.0143 (5)	0.0007 (4)	0.0019 (4)	0.0007 (4)
C18	0.0132 (5)	0.0163 (5)	0.0147 (5)	0.0004 (4)	0.0007 (4)	−0.0002 (4)
C19	0.0147 (5)	0.0197 (5)	0.0153 (5)	−0.0012 (4)	0.0021 (4)	−0.0024 (4)
C20	0.0157 (5)	0.0172 (5)	0.0154 (5)	0.0017 (4)	0.0003 (4)	−0.0013 (4)
C21	0.0169 (5)	0.0207 (5)	0.0197 (6)	−0.0020 (5)	0.0012 (4)	−0.0040 (5)
C22	0.0212 (6)	0.0226 (6)	0.0169 (6)	−0.0003 (5)	−0.0013 (5)	−0.0046 (5)
C23	0.0221 (6)	0.0228 (6)	0.0139 (6)	0.0024 (5)	0.0011 (4)	0.0003 (4)
C24	0.0173 (5)	0.0205 (5)	0.0155 (6)	0.0001 (4)	0.0018 (4)	0.0004 (4)
C25	0.0153 (5)	0.0159 (5)	0.0144 (5)	0.0016 (4)	0.0002 (4)	−0.0004 (4)
C26	0.0147 (5)	0.0161 (5)	0.0134 (5)	0.0014 (4)	0.0011 (4)	−0.0002 (4)
C27	0.0146 (5)	0.0169 (5)	0.0132 (5)	0.0000 (4)	0.0019 (4)	0.0006 (4)
C28	0.0165 (5)	0.0283 (6)	0.0230 (6)	−0.0009 (5)	0.0023 (5)	−0.0062 (5)
C29	0.0229 (6)	0.0226 (6)	0.0240 (7)	−0.0045 (5)	0.0055 (5)	−0.0057 (5)
C30	0.0254 (6)	0.0201 (6)	0.0218 (6)	−0.0035 (5)	0.0046 (5)	−0.0013 (5)

### Geometric parameters (Å, °)

**Table:**

C01—C02	1.5161 (17)	C15—C16	1.3956 (16)
C01—C27	1.5201 (15)	C15—H15	0.9500
C01—C28	1.5416 (17)	C16—C17	1.4754 (15)
C01—H01	1.0000	C17—C18	1.3906 (16)
C02—C03	1.3904 (16)	C18—C26	1.4148 (15)
C02—C07	1.4021 (16)	C18—C19	1.5148 (16)
C03—C04	1.3917 (18)	C19—C20	1.5159 (16)
C03—H03	0.9500	C19—C30	1.5419 (17)
C04—C05	1.3895 (17)	C19—H19	1.0000
C04—H04	0.9500	C20—C21	1.3831 (16)
C05—C06	1.3955 (16)	C20—C25	1.4123 (16)
C05—H05	0.9500	C21—C22	1.3966 (17)
C06—C07	1.3939 (17)	C21—H21	0.9500
C06—H06	0.9500	C22—C23	1.3958 (17)
C07—C08	1.4756 (15)	C22—H22	0.9500
C08—C09	1.3924 (15)	C23—C24	1.3915 (17)
C08—C27	1.4142 (16)	C23—H23	0.9500
C09—C17	1.4131 (16)	C24—C25	1.3952 (16)
C09—C10	1.5188 (16)	C24—H24	0.9500
C10—C11	1.5199 (16)	C25—C26	1.4756 (16)
C10—C29	1.5405 (17)	C26—C27	1.3919 (16)
C10—H10	1.0000	C28—H28A	0.9800
C11—C12	1.3864 (16)	C28—H28B	0.9800
C11—C16	1.4035 (17)	C28—H28C	0.9800
C12—C13	1.3950 (17)	C29—H29A	0.9800
C12—H12	0.9500	C29—H29B	0.9800
C13—C14	1.3896 (18)	C29—H29C	0.9800
C13—H13	0.9500	C30—H30A	0.9800
C14—C15	1.3951 (16)	C30—H30B	0.9800
C14—H14	0.9500	C30—H30C	0.9800
			
C02—C01—C27	101.95 (9)	C18—C17—C09	120.18 (10)
C02—C01—C28	110.87 (9)	C18—C17—C16	131.61 (10)
C27—C01—C28	112.83 (10)	C09—C17—C16	108.19 (10)
C02—C01—H01	110.3	C17—C18—C26	119.89 (10)
C27—C01—H01	110.3	C17—C18—C19	129.52 (10)
C28—C01—H01	110.3	C26—C18—C19	110.59 (10)
C03—C02—C07	120.61 (11)	C18—C19—C20	102.11 (9)
C03—C02—C01	128.25 (11)	C18—C19—C30	112.34 (10)
C07—C02—C01	111.01 (10)	C20—C19—C30	111.02 (10)
C02—C03—C04	119.03 (11)	C18—C19—H19	110.4
C02—C03—H03	120.5	C20—C19—H19	110.4
C04—C03—H03	120.5	C30—C19—H19	110.4
C05—C04—C03	120.59 (11)	C21—C20—C25	120.82 (11)
C05—C04—H04	119.7	C21—C20—C19	128.64 (10)
C03—C04—H04	119.7	C25—C20—C19	110.51 (10)
C04—C05—C06	120.64 (12)	C20—C21—C22	119.09 (11)
C04—C05—H05	119.7	C20—C21—H21	120.5
C06—C05—H05	119.7	C22—C21—H21	120.5
C07—C06—C05	119.01 (11)	C23—C22—C21	120.30 (11)
C07—C06—H06	120.5	C23—C22—H22	119.8
C05—C06—H06	120.5	C21—C22—H22	119.8
C06—C07—C02	120.09 (10)	C24—C23—C22	120.90 (11)
C06—C07—C08	131.50 (11)	C24—C23—H23	119.6
C02—C07—C08	108.34 (10)	C22—C23—H23	119.6
C09—C08—C27	120.21 (10)	C23—C24—C25	119.04 (11)
C09—C08—C07	131.59 (11)	C23—C24—H24	120.5
C27—C08—C07	108.19 (10)	C25—C24—H24	120.5
C08—C09—C17	119.62 (11)	C24—C25—C20	119.81 (11)
C08—C09—C10	129.86 (10)	C24—C25—C26	131.77 (10)
C17—C09—C10	110.52 (9)	C20—C25—C26	108.39 (10)
C09—C10—C11	101.95 (9)	C27—C26—C18	119.88 (10)
C09—C10—C29	112.54 (10)	C27—C26—C25	132.24 (10)
C11—C10—C29	110.72 (9)	C18—C26—C25	107.88 (10)
C09—C10—H10	110.5	C26—C27—C08	119.80 (10)
C11—C10—H10	110.5	C26—C27—C01	129.68 (10)
C29—C10—H10	110.5	C08—C27—C01	110.44 (10)
C12—C11—C16	120.66 (11)	C01—C28—H28A	109.5
C12—C11—C10	128.45 (11)	C01—C28—H28B	109.5
C16—C11—C10	110.75 (10)	H28A—C28—H28B	109.5
C11—C12—C13	118.97 (12)	C01—C28—H28C	109.5
C11—C12—H12	120.5	H28A—C28—H28C	109.5
C13—C12—H12	120.5	H28B—C28—H28C	109.5
C14—C13—C12	120.66 (11)	C10—C29—H29A	109.5
C14—C13—H13	119.7	C10—C29—H29B	109.5
C12—C13—H13	119.7	H29A—C29—H29B	109.5
C13—C14—C15	120.62 (11)	C10—C29—H29C	109.5
C13—C14—H14	119.7	H29A—C29—H29C	109.5
C15—C14—H14	119.7	H29B—C29—H29C	109.5
C14—C15—C16	118.91 (11)	C19—C30—H30A	109.5
C14—C15—H15	120.5	C19—C30—H30B	109.5
C16—C15—H15	120.5	H30A—C30—H30B	109.5
C15—C16—C11	120.16 (11)	C19—C30—H30C	109.5
C15—C16—C17	131.30 (11)	H30A—C30—H30C	109.5
C11—C16—C17	108.46 (10)	H30B—C30—H30C	109.5
			
C27—C01—C02—C03	−178.33 (12)	C11—C16—C17—C18	178.54 (12)
C28—C01—C02—C03	−57.99 (16)	C15—C16—C17—C09	−176.37 (12)
C27—C01—C02—C07	−2.60 (12)	C11—C16—C17—C09	0.28 (13)
C28—C01—C02—C07	117.74 (11)	C09—C17—C18—C26	3.09 (16)
C07—C02—C03—C04	−0.37 (18)	C16—C17—C18—C26	−174.99 (11)
C01—C02—C03—C04	175.00 (11)	C09—C17—C18—C19	−176.57 (11)
C02—C03—C04—C05	−1.24 (18)	C16—C17—C18—C19	5.3 (2)
C03—C04—C05—C06	1.61 (19)	C17—C18—C19—C20	−173.01 (11)
C04—C05—C06—C07	−0.33 (18)	C26—C18—C19—C20	7.30 (12)
C05—C06—C07—C02	−1.26 (17)	C17—C18—C19—C30	67.99 (15)
C05—C06—C07—C08	−177.68 (11)	C26—C18—C19—C30	−111.70 (11)
C03—C02—C07—C06	1.63 (18)	C18—C19—C20—C21	176.85 (11)
C01—C02—C07—C06	−174.48 (10)	C30—C19—C20—C21	−63.23 (16)
C03—C02—C07—C08	178.80 (11)	C18—C19—C20—C25	−5.03 (12)
C01—C02—C07—C08	2.70 (13)	C30—C19—C20—C25	114.90 (11)
C06—C07—C08—C09	−3.7 (2)	C25—C20—C21—C22	0.88 (17)
C02—C07—C08—C09	179.52 (12)	C19—C20—C21—C22	178.83 (11)
C06—C07—C08—C27	175.11 (12)	C20—C21—C22—C23	0.61 (18)
C02—C07—C08—C27	−1.63 (13)	C21—C22—C23—C24	−0.82 (18)
C27—C08—C09—C17	−5.30 (17)	C22—C23—C24—C25	−0.48 (18)
C07—C08—C09—C17	173.44 (11)	C23—C24—C25—C20	1.95 (17)
C27—C08—C09—C10	173.82 (11)	C23—C24—C25—C26	179.84 (12)
C07—C08—C09—C10	−7.4 (2)	C21—C20—C25—C24	−2.18 (17)
C08—C09—C10—C11	177.42 (12)	C19—C20—C25—C24	179.53 (10)
C17—C09—C10—C11	−3.40 (12)	C21—C20—C25—C26	179.47 (10)
C08—C09—C10—C29	−63.93 (16)	C19—C20—C25—C26	1.18 (12)
C17—C09—C10—C29	115.25 (11)	C17—C18—C26—C27	−6.66 (16)
C09—C10—C11—C12	179.26 (12)	C19—C18—C26—C27	173.06 (10)
C29—C10—C11—C12	59.33 (17)	C17—C18—C26—C25	173.32 (10)
C09—C10—C11—C16	3.59 (13)	C19—C18—C26—C25	−6.95 (12)
C29—C10—C11—C16	−116.35 (11)	C24—C25—C26—C27	5.5 (2)
C16—C11—C12—C13	0.69 (18)	C20—C25—C26—C27	−176.45 (12)
C10—C11—C12—C13	−174.61 (12)	C24—C25—C26—C18	−174.51 (12)
C11—C12—C13—C14	0.63 (19)	C20—C25—C26—C18	3.56 (12)
C12—C13—C14—C15	−1.14 (19)	C18—C26—C27—C08	4.24 (16)
C13—C14—C15—C16	0.32 (18)	C25—C26—C27—C08	−175.74 (11)
C14—C15—C16—C11	1.00 (17)	C18—C26—C27—C01	−172.36 (11)
C14—C15—C16—C17	177.32 (11)	C25—C26—C27—C01	7.7 (2)
C12—C11—C16—C15	−1.52 (18)	C09—C08—C27—C26	1.74 (17)
C10—C11—C16—C15	174.55 (10)	C07—C08—C27—C26	−177.27 (10)
C12—C11—C16—C17	−178.61 (11)	C09—C08—C27—C01	178.95 (10)
C10—C11—C16—C17	−2.54 (13)	C07—C08—C27—C01	−0.06 (13)
C08—C09—C17—C18	2.87 (17)	C02—C01—C27—C26	178.41 (11)
C10—C09—C17—C18	−176.40 (10)	C28—C01—C27—C26	59.45 (16)
C08—C09—C17—C16	−178.63 (10)	C02—C01—C27—C08	1.55 (12)
C10—C09—C17—C16	2.09 (13)	C28—C01—C27—C08	−117.41 (11)
C15—C16—C17—C18	1.9 (2)		
